# A Rare Life-Threatening Presentation of Shoulder Pain: Cervical Epidural Hematoma

**DOI:** 10.7759/cureus.33510

**Published:** 2023-01-08

**Authors:** Takshak Shankar, Vempalli Nagasubramanyam, Archana Bairwa, Ashwani Pundir

**Affiliations:** 1 Emergency Medicine, All India Institute of Medical Sciences, Rishikesh, IND

**Keywords:** bilateral shoulder pain, spontaneous cervical epidural hematoma, cervical epidural hematoma, atypical presentation shoulder pain, shoulder joint pain

## Abstract

Shoulder pain is a common complaint of patients presenting to emergency department. Various conditions, intrinsic and extrinsic to the shoulder, can result in shoulder pain. Some of these extrinsic conditions can pose a threat to life. We present a case of a young, previously healthy male who initially had bilateral shoulder pain, later developed quadriparesis, and was ultimately diagnosed with a spontaneous cervical epidural hematoma. He underwent an emergency C7-T1 laminectomy with hematoma evacuation and had a full recovery. Cervical epidural hematoma is a rare surgical emergency where timely diagnosis and treatment are crucial.

## Introduction

Shoulder pain is a common musculoskeletal condition. Several intrinsic and extrinsic conditions can result in shoulder pain, and they often have overlapping presentations. Thus, arriving at the correct diagnosis requires a meticulous clinical examination and a battery of investigations. Since shoulder pain can also result from various life-threatening causes extrinsic to the shoulder, these conditions must be actively ruled out in every patient with shoulder pain [1,2]. Here, we present a case of a young, previously healthy male who complained of bilateral shoulder pain for two days, later developed quadriparesis, and was ultimately diagnosed with a spontaneous cervical epidural hematoma.

## Case presentation

A young male in his 20s, previously healthy, presented to emergency department complaining of pain in both shoulders for two days and weakness in all four limbs for one day. The shoulder pain was a vaguely localized pain on the posterior aspect of both shoulders, and it was more on the left side. It was mild-to-moderate in intensity, and all movements at the shoulder joint were painful, with the pain not aggravated by any particular movement. The patient had recently joined a gym, and since the pain developed a few hours after exercising his shoulders, it was initially attributed to delayed onset muscle soreness. The next day the pain in his shoulders increased, and he developed acute onset weakness of all four limbs, which was gradually progressive, with bladder and bowel incontinence. He was rushed to a local physician, who referred him to our hospital for further management. En route to our hospital, the weakness in the upper limb began to improve gradually, but the lower limb weakness was static. He denied any history of drug abuse, fever, trauma, loss of consciousness, or seizures and was on no antiplatelet or anticoagulant drugs.

On examination, the patient was conscious and oriented to time, place, and person. He was afebrile with a pulse rate of 88/min, blood pressure of 110/70 mmHg, respiratory rate of 18 breaths/min, and SpO_2_ of 100% on room air. CNS examination was significant for increased tone in both lower limbs with normal tone in both upper limbs. The power was 4/5 in both upper limbs at the shoulder, elbow, and wrist joints, while the power in both the lower limbs was 2/5 at the hip joint, 3/5 at the knee joint, 4/5 at the ankle joint, and 4/5 at the extensor hallucis longus. The bilateral knee and supinator deep tendon reflexes were brisk, and ankle clonus was present. Pupils were bilaterally normal in size, equal, and normally reacting to light. There was no midline cervical tenderness. Sensory examination was normal. The rest of the systemic examination was within normal limits. The patient’s blood investigations on arrival at emergency department are tabulated in Table 1.

**Table 1:** The blood investigations of the patient at emergency department presentation. INR: international normalized ratio; aPTT: activated partial thromboplastin time; ALT: alanine transaminase; AST: aspartate transaminase

Investigation	Result	Reference values
Hemoglobin (mg/dL)	14.3	13-17
Platelet count (thousand/cu mm)	2,26,000	1,50,000-4,50,000
Serum creatinine (mg/dL)	0.62	0.7-1.2
Blood urea (mg/dL)	13	13-43
INR	1.02	0.90-1.10
aPTT (s)	36	35
ALT (U/L)	27	0-50
AST (U/L)	25	0-50
Total bilirubin (mg/dL)	0.91	0.3-1.2
Direct bilirubin (mg/dL)	0.14	0-0.2

**Figure 1:**
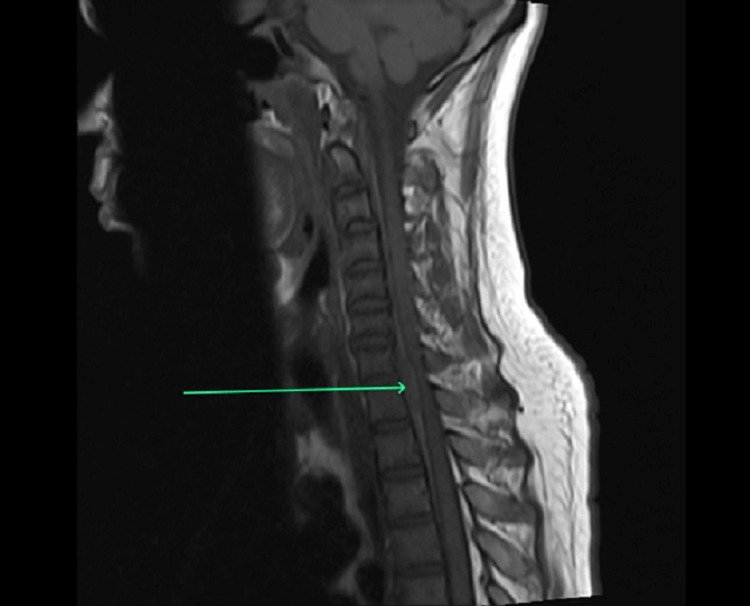
T1-weighted image of the sagittal section of the cervical spinal cord showing a well-defined extradural extramedullary isointense lesion along the anterior surface of the spinal cord extending from mid-C7 to mid-T1 vertebral levels (arrow).

**Figure 2:**
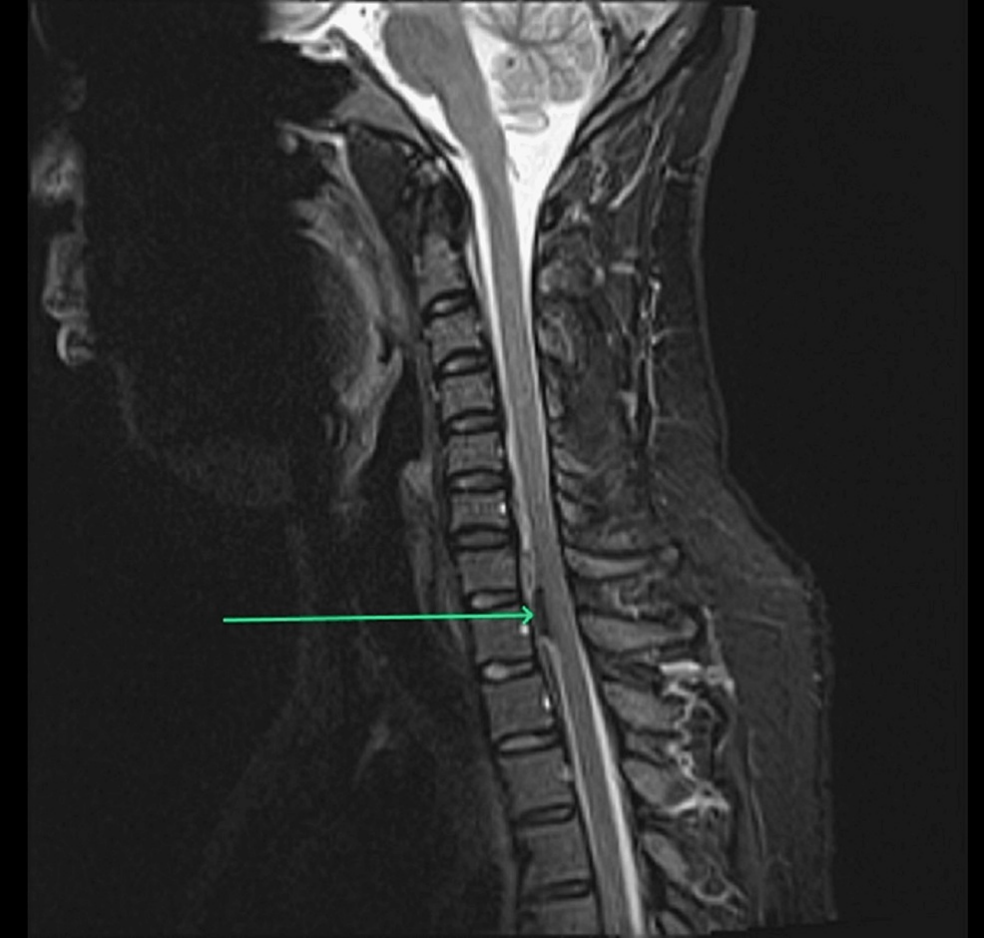
T2-weighted image of the sagittal section of the cervical spinal cord showing a well-defined extradural extramedullary hypointense lesion along the anterior surface of the spinal cord extending from mid-C7 to mid-T1 vertebral levels (arrow).

Additionally, the patient’s bleeding time and clotting time were within the normal range. An MRI cervical spine with whole spine screening was suggestive of a well-defined, extradural, extramedullary lesion which was T1 isointense and T2/short-tau inversion recovery (STIR) hypointense, seen along the anterior surface of the spinal cord which was extending from C7-T1 vertebral level with compression of the spinal cord (Figures 1, 2).

Hence, a diagnosis of spontaneous cervical epidural hematoma causing cord compression was made. The patient underwent an emergency C7-T1 laminectomy with hematoma evacuation. Around 24 h postoperatively, the power improved to 4/5 in all four limbs. The patient was discharged on day 10 with full power in all four limbs and continent bladder and bowel.

## Discussion

Shoulder pain is a common musculoskeletal complaint of patients presenting to emergency department. Shoulder pain can result from either intrinsic disorders of the shoulder or referred pain. The intrinsic disorders of the shoulder comprise injuries and degeneration or inflammation of the joint, tendons, ligaments, or periarticular structures [1]. Shoulder pain can also be the result of certain dangerous extrinsic causes, which include but are not limited to cervical nerve root compression, brachial plexus lesions, myocardial ischemia, axillary vein thrombosis, and irritation of the diaphragm secondary to splenic injury, ruptured ectopic pregnancy, or a perforated viscus. Intrinsic disorders of the shoulder usually present with any or a combination of pain resulting from certain movements, stiffness, weakness, and instability. A poorly localized shoulder pain with a normal shoulder examination should raise concerns of an extrinsic pathology causing the pain [2].

Injuries and overload of the skeletal muscle are common in sports, with delayed onset muscle soreness (DOMS) describing an entity of ultrastructural muscle damage caused by eccentric or unfamiliar forms of exercise. The clinical manifestations of DOMS start 6-12 h after exercising, gradually increasing to a peak at 48-72 h and then decreasing until they disappear after five to seven days. DOMS results in a painful restriction of movement, decreased force capacities, stiffness, swelling, and dysfunction of adjacent joints [3-5].

A spinal epidural hematoma is typically considered a surgical emergency [6]. This is a rare condition with a reported incidence of zero to one per 1,00,000 per year, although, with the wider usage of MRI, the incidence has increased in recent years [7-9]. It can occur secondary to trauma, tumor, coagulopathy, vascular malformation, cavernous angioma, or can be spontaneous [7,9-11]. The age of presentation of spontaneous spinal epidural hematoma (SSEH) shows two peaks in the second and the seventh decades [9]. The common location of SSEH is at the C6 and the T12 levels. Most of these hematomas are around 3.6 vertebral levels long [6]. They are usually located dorsal to the spinal cord owing to the Hoffmann ligaments, which connect the posterior longitudinal ligament to the ventral dura [12].

Spinal epidural hematomas of the cervical spine have an acute presentation and are usually spontaneous. Current literature supports the hypothesis of both arterial and venous origins as the cause of bleeding in SSEH [6,13]. The widely accepted theory is that these hematomas are venous in origin as the spinal epidural veins are valveless and thus are not protected from pressure changes in the thorax or abdomen [14]. However, this venous theory does not seem plausible in the cervical region, owing to the rapidity of development and also because the venous pressure in the cervical region is lower than even the intrathecal pressure [6]. The hypothesis proposed by Beatty and Winston says that the free anastomotic arteries traversing the epidural space are the source of arterial bleeding, which forms the hematoma [15].

The clinical presentation of SSEH depends on the location and degree of cord compression. Usually, patients present with severe back or neck pain with a radicular component and neurologic deficits. The severity of sensorimotor deficit has a prognostic significance as patients with some residual function are more likely to show complete recovery [6,16-18].

MRI is the investigation of choice for diagnosis, although contrast may be required to differentiate it from an epidural abscess [12,18]. In the initial 24 h, the epidural hematoma is iso-intense to the cord on T1-weighted (T1W) sequences and is hyperintense or heterogenous on T2-weighted (T2W) sequences. By 48 h, it is hyperintense on both T1W and T2W sequences [8,10,12].

The preferred treatment for SSEH is a decompressive laminectomy with hematoma evacuation, although the use of conservative methods is gradually increasing. However, the use of conservative methods poses the risks of worsening neurological deficits and increasing hematoma size. The American Spinal Cord Injury Association (ASIA) scoring system determines the need for operative care. Early surgeries are associated with better outcomes [19,20].

Our patient complained of vaguely localized bilateral shoulder pain for a day before the development of neurological deficits. Shoulder pain can result from various intrinsic and extrinsic conditions, some of which are life-threatening. Since shoulder pain is a common complaint of patients visiting the emergency department and time constraints often limit emergency evaluation, it is prudent that life-threatening extrinsic causes of shoulder pain be ruled out in every patient whose pain is not altered by shoulder movements.

## Conclusions

Shoulder pain results from multiple conditions and reaching an accurate diagnosis may require a battery of investigations. Often, all of these investigations can’t be performed in the emergency setting, but life-threatening extrinsic and intrinsic causes must be ruled out in every patient as far as possible. Patient education is also extremely important here as some patients may delay seeking medical attention or be lost to follow-up, delaying diagnosis and timely interventions.

## References

[1] Anderson B (2006). Office Orthopedics for Primary Care: Treatment.

[2] Vaughan A (2022). Evaluation of the adult with shoulder complaints. https://www.uptodate.com/contents/evaluation-of-the-adult-with-shoulder-complaints.

[3] Hotfiel T, Freiwald J, Hoppe MW (2018). Advances in delayed-onset muscle soreness (DOMS): part I: pathogenesis and diagnostics. Sportverletz Sportschaden.

[4] Pearcey GE, Bradbury-Squires DJ, Kawamoto JE, Drinkwater EJ, Behm DG, Button DC (2015). Foam rolling for delayed-onset muscle soreness and recovery of dynamic performance measures. J Athl Train.

[5] Valle X, Til L, Drobnic F, Turmo A, Montoro JB, Valero O, Artells R (2014). Compression garments to prevent delayed onset muscle soreness in soccer players. Muscles Ligaments Tendons J.

[6] Mueller-Wohlfahrt HW, Haensel L, Mithoefer K (2013). Terminology and classification of muscle injuries in sport: the Munich consensus statement. Br J Sports Med.

[7] Baek BS, Hur JW, Kwon KY, Lee HK (2008). Spontaneous spinal epidural hematoma. J Korean Neurosurg Soc.

[8] Vázquez-Barquero A, Abascal F, García-Valtuille R, Pinto JI, Figols FJ, Cerezal L (2000). Chronic nontraumatic spinal epidural hematoma of the lumbar spine: MRI diagnosis. Eur Radiol.

[9] Gala FB, Aswani Y (2016). Imaging in spinal posterior epidural space lesions: a pictorial essay. Indian J Radiol Imaging.

[10] Fukui MB, Swarnkar AS, Williams RL (1999). Acute spontaneous spinal epidural hematomas. AJNR Am J Neuroradiol.

[11] Lan T, Chen Y, Yang XJ (2015). Spontaneous spinal epidural haematoma. J Orthop Translat.

[12] Avrahami E, Tadmor R, Ram Z, Feibel M, Itzhak Y (1989). MR demonstration of spontaneous acute epidural hematoma of the thoracic spine. Neuroradiology.

[13] Gopalkrishnan CV, Dhakoji A, Nair S (2012). Spontaneous cervical epidural hematoma of idiopathic etiology: case report and review of literature. J Spinal Cord Med.

[14] Holtås S, Heiling M, Lönntoft M (1996). Spontaneous spinal epidural hematoma: findings at MR imaging and clinical correlation. Radiology.

[15] Beatty RM, Winston KR (1984). Spontaneous cervical epidural hematoma. A consideration of etiology. J Neurosurg.

[16] Salehpour F, Mirzaei F, Kazemzadeh M, Alavi SA (2018). Spontaneous epidural hematoma of cervical spine. Int J Spine Surg.

[17] Liao CC, Lee ST, Hsu WC, Chen LR, Lui TN, Lee SC (2004). Experience in the surgical management of spontaneous spinal epidural hematoma. J Neurosurg.

[18] Bruyn GW, Bosma NJ (1976). Spinal extradural hematoma. Injuries of the Spine and Spinal Cord. Part 2.

[19] Raasck K, Habis AA, Aoude A, Simões L, Barros F, Reindl R, Jarzem P (2017). Spontaneous spinal epidural hematoma management: a case series and literature review. Spinal Cord Ser Cases.

[20] Fiani B, Jarrah R, Fiani NJ, Runnels J (2021). Spontaneous cervical epidural hematoma: insight into this occurrence with case examples. Surg Neurol Int.

